# The Mare: A Pertinent Model for Human Assisted Reproductive Technologies?

**DOI:** 10.3390/ani11082304

**Published:** 2021-08-04

**Authors:** Achraf Benammar, Emilie Derisoud, François Vialard, Eric Palmer, Jean Marc Ayoubi, Marine Poulain, Pascale Chavatte-Palmer

**Affiliations:** 1 Université Paris-Saclay, UVSQ, INRAE, BREED, 78350 Jouy-en-Josas, France; a.benammar@hopital-foch.com (A.B.); emilie.derisoud@inrae.fr (E.D.); francois.vialard@uvsq.fr (F.V.); jm.ayoubi@hopital-foch.com (J.M.A.); marine.poulain@hopital-foch.com (M.P.); 2 Ecole Nationale Vétérinaire d’Alfort, BREED, 94700 Maisons-Alfort, France; 3 Department of Gynaecology and Obstetrics, Foch Hospital, 92150 Suresnes, France; 4 Académie d’Agriculture de France, 75007 Paris, France; epcryozoo@gmail.com

**Keywords:** oocyte, embryo, intracytoplasmic sperm injection, ovum pick-up, maternal age, obesity, exercise, in vitro maturation

## Abstract

**Simple Summary:**

Artificial reproduction techniques (ART) are used widely in human medicine to overcome infertility, with about one in seven couples being concerned in the Western world. Due to ethical concerns, animal models are needed to develop new methodologies. Although laboratory animals are seminal in this context, they have a short lifespan and are usually fertile. Horses are long-lived domestic animals that are bred until old age, often after they have had a career being used for equestrian activities. Their reproductive functions become altered after 20 years, in a similar way to humans, although there is no menopause *per se* in horses. There is also a concern for rising overweight and obesity concerns in these species. In addition, embryo transfer and ART are developed to overcome infertility, as for humans. This review details similarities and differences in the reproductive cycle, ART, and fertility concerns in women and mares and discusses the opportunity of using the horse as an appropriate model for ART in humans.

**Abstract:**

Although there are large differences between horses and humans for reproductive anatomy, follicular dynamics, mono-ovulation, and embryo development kinetics until the blastocyst stage are similar. In contrast to humans, however, horses are seasonal animals and do not have a menstrual cycle. Moreover, horse implantation takes place 30 days later than in humans. In terms of artificial reproduction techniques (ART), oocytes are generally matured in vitro in horses because ovarian stimulation remains inefficient. This allows the collection of oocytes without hormonal treatments. In humans, in vivo matured oocytes are collected after ovarian stimulation. Subsequently, only intra-cytoplasmic sperm injection (ICSI) is performed in horses to produce embryos, whereas both in vitro fertilization and ICSI are applied in humans. Embryos are transferred only as blastocysts in horses. In contrast, four cells to blastocyst stage embryos are transferred in humans. Embryo and oocyte cryopreservation has been mastered in humans, but not completely in horses. Finally, both species share infertility concerns due to ageing and obesity. Thus, reciprocal knowledge could be gained through the comparative study of ART and infertility treatments both in woman and mare, even though the horse could not be used as a single model for human ART.

## 1. Introduction 

Animal breeding has been performed by humans since prehistoric times. Selective breeding really started in the early 18th century in the UK with Sir Robert Bakewell, who developed objective selection through accurate recording of animal performance and progeny testing [1]. Criteria used to select domestic animals could be classified in different categories: (1) those necessary for human feeding, (2) those necessary for their physical capacities, and (3) those related to aesthetic or behavioral properties. In the first category, product quantity and/or quality, according to species (meat, milk, wool, eggs…), has been the selection criteria. Associated with production and rentability, fertility has also been positively selected because infertile animals were usually culled. With genetic progress being based on reproductive speed, young and fertile animals are used as the selection base. In the second category, the initial objective was to provide labor, which, when automobiles and tractors were introduced, declined in popularity. The third category concerns a specific behavior (specific gait for example) or a particularly praised esthetical trait. In this case, selection will be based on young animals as soon as the phenotype is known, or, like the other categories, as far as the genotype can predict the phenotype of these animals, on the individual’s genotype. Thus, in most selection processes, aged and infertile animals are not considered.

With these selection objectives, in cattle, assisted reproductive technologies (ART) are used commonly to produce embryos, with an increasing number of embryos produced in vitro each year worldwide (>10^6^ in 2019) whereas the number of in vivo derived embryos declines steadily (around 4 × 10^5^ in 2019) [2], with younger animals, even prepubertal, being used to speed up genetic progress in combination with genomic selection. In horses, however, horse meat consumption is not the main focus of horse production. Most horses are bred for sport or leisure, and depending on their use, will reproduce after they have achieved their career, whether as an athlete (i.e., in racing, endurance, show jumping, dressage, steeplechase) or as a leisure horse (i.e., pony and riding clubs, equestrian tourism). Selection for reproduction is based on performance and, depending on the breed, use, and geographical location, genetic indices may be or not calculated and used for selection. Many male horses are gelded at an early age (often 2 years) for management, so that only highly valued animals are kept for breeding. Thus, both males and females may be used for reproduction until they become elderly. In these species, when allowed by the breed’s studbook, ART is used to breed elite infertile and/or older animals, even though genetic progress is hampered when breeding older animals [3].

Regarding humans, it has been estimated that 15% of couples in industrial countries are infertile, with a frequency that continuously increases due to environmental problems and delayed pregnancy [4,5]. Indeed, the chance to conceive per cycle, for women between 20 and 30 years old, has been estimated at 21–28% per cycle and decreases with maternal age. In most of the Western world, women’s age when they have their first child is increasing [4]. To improve human ART, new technologies and research programs are necessary. Due to ethical concerns, there are many limitations to experimentation using human embryos and the evaluation of new technologies is often impossible. Thus, animal models are seminal to progress in human ART. Studying gametogenesis, the mouse model seems the more appropriate, but there are two main limitations: (1) the efficiency of gametogenesis is dramatically different when compared to human and (2) environmental modelling is quite limited in many situations, while in terms of fertilization, the mouse model remains the best model [6]. For the study of embryo development until implantation, the mouse is definitely very different from all other mammals. Appropriate models are still being discussed, with rabbit development being very close to that of human, indicating that these species should be further employed as a model for human ART [7], while the wide use of ART in cattle throughout the world [2] provides insight in short and long term environmental effects of ART [6,8]. The horse has been pointed out as an appropriate model for follicular development and oocyte ageing [9,10], but may be more widely pertinent as a model for humans.

The aim of this review is to evaluate the horse as a model to study human reproduction and particularly to improve ART considering that (1) selection is not based on reproduction capacity [3,11], (2) infertility seems to impact a large number of stallions and elderly mares [12], (3) reduced activity in leisure horses leads to frequent obesity [13,14,15], and (4) pregnancy occurs after sports career retirement at middle age [16].

## 2. Comparative Anatomical, Physiological and Pathological Aspects of Reproduction in Mares and Women

### 2.1. Anatomical Considerations: Uterus and Ovaries

The uterus is the maternal organ that receives the embryo. It ensures, through its secretions, its development until implantation. Uterine anatomy differs among mammalian species and is adapted to their reproductive biology, such as trans-uterine migration of blastocysts and litter size. The uterine tissue is composed of an external muscular tunic (myometrium) surrounding an internal glandular layer, the endometrium. The human uterus is simple with one large uterine body, 7 cm long, without uterine horns while the horse uterus is bicornuate with a 5–8 cm straight cervix and a 20 cm long uterine body communicating with two uterine horns about 30 cm long.

Ovarian development occurs during fetal life both in humans and horses, with initiation of meiosis until meiotic prophase taking place in the first half of gestation and folliculogenesis occurring roughly from mid pregnancy until puberty (around 12 years in humans vs. 1 year of age in horses) [17]. In women, the fertility capital, represented by her follicular reserve, is definitively constituted during fetal life and estimated at approximately ± 7 million at 20 weeks gestation [18]. In comparison, a smaller number of primordial follicles (around 36,000 with a high variability among individuals) is observed in 2–4 years old (adult) mares [19].

**Figure 1:**
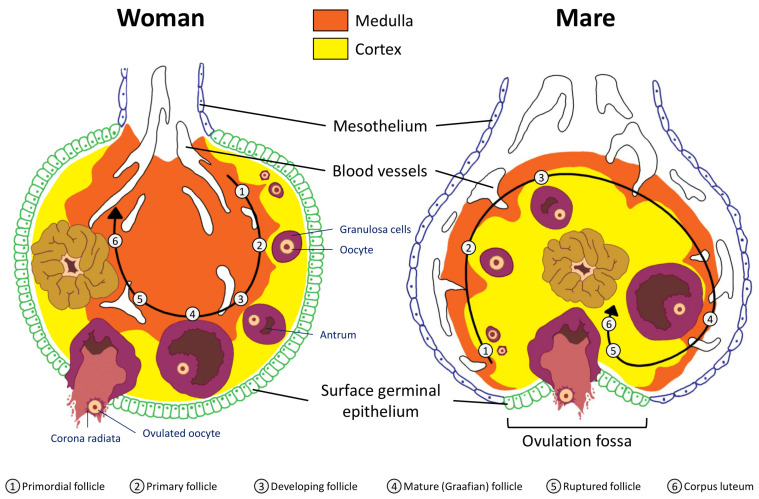
Comparative ovarian anatomy and folliculogenesis in woman and mare (inspired from [20]).

The woman’s ovary is a smooth organ, measuring about 4 cm long, 2 cm wide and 1 cm thick. The outer stromal connective tissue, called cortex, that encloses follicles, is located below the surface germinal epithelium and the albuginea, whereas the central connective tissue, called medulla, is composed of a hilar zone (containing vessels, nerves…), a parenchymatous zone with loose connective tissue crossed by vessels in relation with the cortex and the rete ovarii (the hilum) (Figure 1). The equine ovary is approximately the size of a chicken egg (around 5 cm in length and 3 cm in width), with a kidney-shape structure. The internal structure consists of a central “ovarian cortex” with follicles surrounded, except for the area of the ovarian fossa, by a very thin tissue corresponding to the “medulla” in other species and humans. The ovarian fossa, in the concave area of the ovary, is the only place where ovulations can occur. The rest of the surface of the gonad is covered by the visceral peritoneum [20].

### 2.2. Folliculogenesis

Folliculogenesis is a long process in which regulatory mechanisms are not well known.

**Figure 2:**
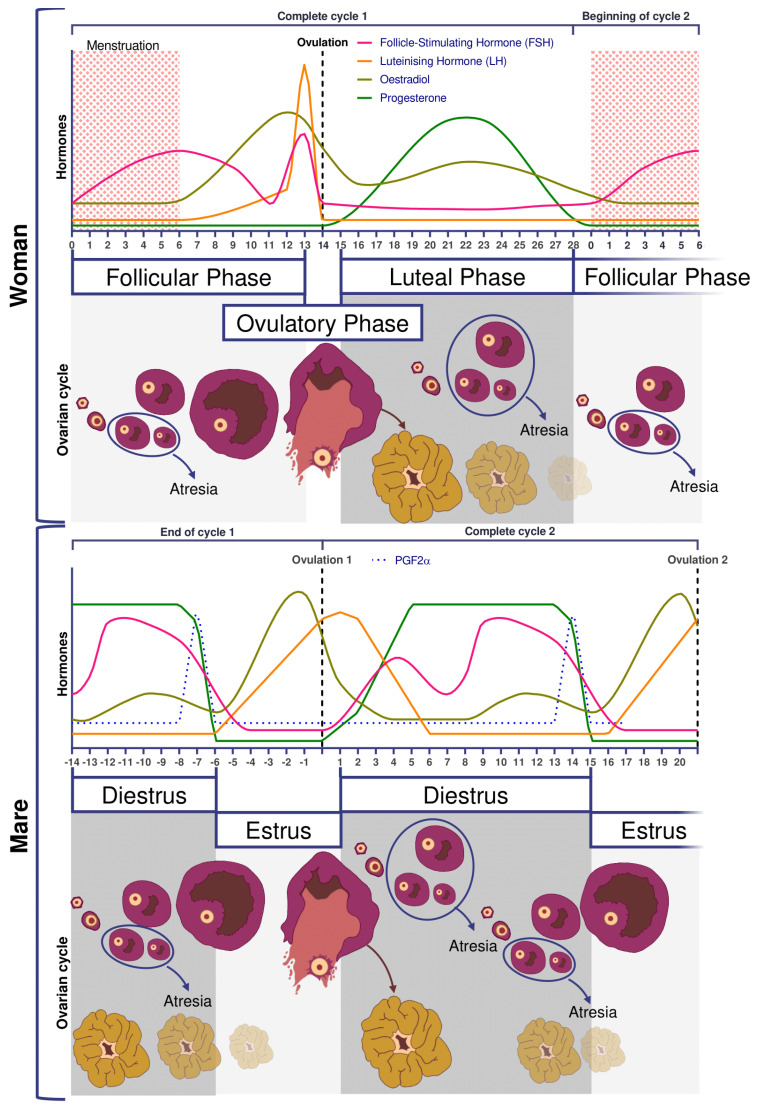
Comparative ovarian and hormonal cycles in women and mares.

Reproductive and ovarian cycles of women and mares are represented in Figure 2. Conventionally, in women, the cycle lasts 28 days [21], with the first day of the menstrual cycle being the first day of menstruation. Conversely, in mares, the cycle starts on the day of ovulation. An unfertilized mare cycle lasts on average 22 days, including 5–7 days of estrus at the end which ovulation takes place. Without fertilization, luteolysis begins after 12 days post ovulation [20]. In addition, the mare is a seasonal mammal with resumption of cyclicity associated with increased day length [22,23].

In women and mares, at puberty, follicular growth resumes due to the maturation of the hypothalamic-pituitary axis which leads to the secretion of Follicle Stimulating Hormone (FSH), and Luteinizing Hormone (LH) and the stimulation of follicles ≥2 mm in a cyclic manner (reviewed in [21] for humans and [24] for horses). Regularly, a group of primordial follicles starts growing to reach the pre-antral stage (0.1–0.2 mm). As also described in horses, this first phase of follicular growth is independent of gonadotropin support [25]. Once a 0.2–0.4 mm diameter is reached, the antrum begins to develop and follicles become responsive to gonadotropins [25,26]. Follicular depletion begins from fetal life and continues during childhood and throughout reproductive female life in both species [27]. The entire duration of human folliculogenesis is still unknown in both species but has been estimated, from the primordial phase to the preovulatory phase, to span over 200 days in humans [28].

Follicular dynamics are remarkably comparable between humans and horses, with the final dominance of one follicle [26,29]. In both species, follicular growth is due to more complex mechanisms through successive follicular waves (2–3 in horses) that may or may not lead to the development of a dominant follicle that will become ovulatory or not [29,30]. These waves can be described as minor (without appearance of a dominant follicle), major (appearance of dominant follicle ≥10 mm diameter), or as alternating between one and the other in a way that seems random [29,31]. A single dominant follicle is selected in the middle of the major follicular phase and deviates from the growth trajectory of the other follicles until it ovulates, while all other subordinate follicles regress (also known as follicular deviation) [32].

In women, FSH concentrations are low during the luteal phase and rise at the beginning of the follicular phase. On day 7, FSH level begins to decrease (i.e., end of the FSH window), leading to the dominance of only one follicle, generally the biggest one, whose growth is FSH independent [18]. In mares, pronounced FSH surges are observed in the middle of the diestrus.

The diameter of the largest follicle at time of deviation and the maximum diameter of the preovulatory follicle is consistently 2.1 times greater in the mare compared to the woman [31] (around 2 cm in women and >4 cm in mares).

### 2.3. Ovulation

Ovulation is preceded by an increase in plasma concentrations of estradiol, FSH, and LH, beginning slightly before follicular deviation in both mare and woman (Figure 2) [31]. When the dominant ovarian follicle produces enough estradiol (plasma concentrations of total estradiol in the range of 200–300 pg/mL [33] and about 10 times less in horses [34]), this induces a negative retrocontrol on the hypothalamus conducive to a reduction in FSH secretion. The dominant follicle becomes autonomous and the others undergo apoptosis. Hypothalamic kisspeptin and GnRH secretion increase, resulting in a surge in pituitary secretion of LH roughly at the time of peak estradiol concentrations.

Characteristics of the horse include estrous behavior, characterized by sexual attractiveness to stallions and mating behavior in mares. The drop in plasma progesterone concentrations is a prerequisite for the rising estrogen to induce estrus. Estrus can last up to 6–7 days, with ovulation occurring around 24 h before the end of estrus. Among other differences between the two species, there is no FSH peak at the time of ovulation [35], whereas in women the LH surge is associated with small increases in FSH, with a similar time course [31].

In both mares and women, there is usually one ovulation in normal conditions but double ovulations are frequent. Double ovulations are more of a concern in horses as twin pregnancies are considered as pathological as most result in abortion. They occur in about 20% of the cycles in mares, but this differs according to breed [36] and increases significantly with mare’s [37,38] and women’s age [39].

If fertilization does not occur, luteolysis takes place, thus starting a new cycle. In women, luteolysis is not dependent on uterine prostaglandin secretion as hysterectomized women have normal hormonal curves [40]. However, the lack of hCG and so far still undescribed mechanisms will induce luteolysis [41]. In the horse, as for other domestic animal, luteolysis is dependent on the uterine production of prostaglandin a (as reviewed by [42]).

Anovulation is one of the main causes of infertility in women and females of many domestic species. One of the causes of anovulation, common to both the women [43,44] and mares [45,46,47], is luteinized unruptured follicle (LUF), the precise pathophysiology of which has yet to be determined. LUFs are highly repeatable across cycles (79–90%), resulting in recurrent anovulation [48,49] and infertility in humans. This ovulatory dysfunction has been documented to occur in 11–23% of women with normal menstrual cycles [50,51], 13–73% of women with endometriosis [52], and 4–58% of women with unexplained infertility [53,54]. In cycling mares, LUFs, also known as HAFs (Hemorrhagic Anovulatory Follicles), are also present in 5–20% of estrous cycles [46,47,55,56]. A 5% incidence of LUFs has been reported during the early ovulatory season, followed by 20% during the late reproductive season [46,57], and it seems to occur more frequently in older mares.

In primates, including humans, the ovulated eggs adhere with their cumulus mass of follicular cells to the surface of the ovary. The fimbrial end of the tube sweeps across the ovary to retrieve the egg. Entry into the tube is facilitated by muscular movements that bring the fimbriae into contact with the surface of the ovary. Although there is a small negative pressure in the tube in association with muscle contractions, this does not condition the recovery of the oocytes by the tube [58]. In horses, the uterine extremity of the ovary is attached to the uterus near the tip of the uterine horn by the utero-ovarian ligament, which forms the ovarian bursa that faces the ovulation fossa (Figure 1). This structure considerably reduces the risks of ectopic pregnancy, which is extremely rare [59].

### 2.4. Preimplantation Embryos

In humans, the acellular zona pellucida surrounds the ovum at ovulation and remains in place until embryo hatching prior to implantation. In horses, an outer gelatinous layer surrounds the oocyte. It is assumed that it consists of cytoplasmic debris from granulosa cells [60] and it disappears after fertilization so that only the zona pellucida still protects the equine embryo at this time [61,62].

**Figure 3:**
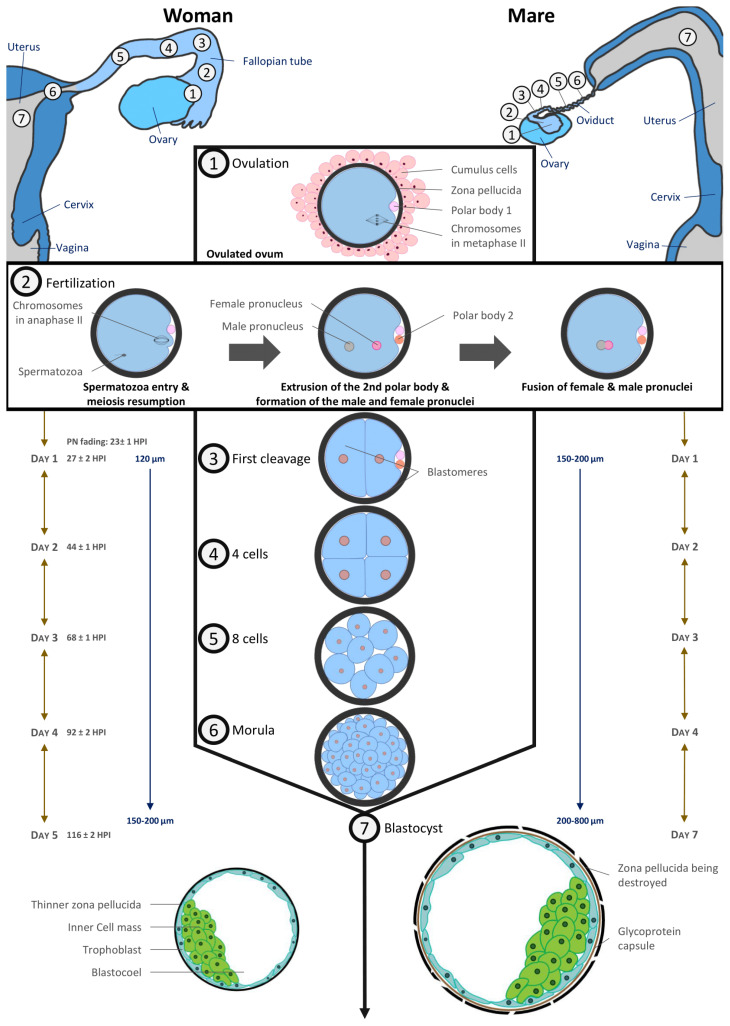
Comparative embryo development in Human and equine (inspired from [20,87]).

In vivo, fertilization takes place in the oviduct. The timing of the different stages of embryo development is depicted in Figure 3 and is very similar between the two species. Fertilized oocytes undergo their first cleavage during the first 24–27 h post fertilization. Next cell divisions occur quickly: the embryo reaches the 4–6 cell stage within 44–48 h and the 16 cell stage within 68–72 h after fertilization [61,63].

The culture of human embryos in time-lapse imaging systems has allowed more recently to estimate more precisely the timing of cell divisions linked to blastocyst formation and embryo potential in terms of implantation [64]. During early cleavages, the human embryo needs more lactate and pyruvate as energetic substrates to develop [65].

Translation of maternal transcripts within the human embryo begins very early: DNA synthesis activity can be detected 9–10 h after insemination [66]. Embryonic genome transcription begins between the 4- and 8-cell cleavage stages, i.e., 2–3 days after fertilization [67]. Earlier embryonic signals can be detected shortly after fertilization. As for humans, at the 4th cell stage (6–8 cell stage), the nucleolus of equine embryos is reorganized and embryonic transcription begins [68].

The size of the equine conceptus does not increase during the early stages of development, remaining on average less than 200 µm [61,69]. At this developmental stage, equine embryo carbohydrate metabolism requires as much pyruvate as glucose [66]. By day 7 post ovulation, the blastocoel is formed, and equine embryos have become blastocysts: blastomeres having differentiated in the inner cell mass regrouped in a compact round zone and the trophectoderm, an epithelium that surrounds the entire embryo [61,62,67]. By day 8, the equine blastocyst cavity is entirely layered by endodermal cells [70].

Embryo development is quite similar in humans with a compaction of embryonic cells on day 4 when the embryo reaches around 16 cells. Blastulation starts on day 5 with the individualization of two distinct cell lines which are trophectoderm cells lining the blastocoelic cavity and inner mass cells. Until the morula stage, cell divisions are said to be reductive because they take place at constant embryonic volume within the zona pellucida (around 120 µm). From day 4, embryo metabolism switches towards the consumption of glucose and amino acids [71,72].

A complex transport system ensures the transport of the fertilized human oocyte to its implantation site, the uterus, by means of three different components, (1) ciliary movement, (2) muscle contractility, and (3) tubal fluid, all of which contribute in varying degrees to efficient tubal transport. Various hormonal and neural factors have been shown to modulate ciliary activity, including adrenergic and cholinergic stimulation, ovarian steroids, prostaglandins, angiotensin II and adrenomedullin. It is difficult to access precise timing data in humans, but it has been described that the first embryonic cleavages occur during transport through the tube. The embryo migrates from the location of the fertilization in ampulla to the isthmus and reaches the uterine cavity at blastocyst stage on day 5. During its migration to the uterus, the zona pellucida prevents the embryo from prematurely adhering to the oviduct rather than traveling to the uterus [73].

The mare is unique in that only fertilized oocytes (developing embryos) enter the uterus, approximately 5.5–6.5 days following ovulation [74,75,76], i.e., at the late morula/early blastocyst stage [61,66,76]. Indeed, prostaglandins E2 production by equine embryos in the oviduct induces oviductal muscle relaxation in the isthmus [77,78,79,80]. As unfertilized oocytes do not produce prostaglandin E2, they are retained in the oviduct for several estrous cycles. These unfertilized oocytes are at the origin of masses in oviducts [81].

In most species, time spent in the oviduct is a prerequisite for full development. This may not be the case in humans as successful pregnancies have occurred in humans following the Estes procedure, in which the ovary is transposed into the uterine cavity in case of tubal infertility [82,83]. Moreover, embryo transfers in the uterine cavity of patients are frequently performed at cleavage stages and some ART centers reported transfers at zygote stage with relatively good chances of pregnancy [84,85]. In equine, embryos have been shown to survive in the uterus as early as four days after fertilization [67].

The blastocyst stage is the last common developmental stage between horses and humans. The human blastocyst implants around 7–10 days post fertilization. The zona pellucida becomes thinner as the blastocyst expands and finally ruptures to allow blastocyst hatching before implantation while the blastocyst is still a hatched blastocyst and the uterus receptive. Conversely, in horses, the blastocyst remains free and continues to grow. Under the influence of uterine contractions, it moves in the mare’s uterus until 16–17 days post ovulation, when uterine edema is such that movement stops. It has been shown that the movement of the embryo is involved in maternal recognition of pregnancy, although the exact signal remains controversial (for review [86]). Implantation begins at 35–40 days after ovulation (for review [87]), and therefore an important part of embryo organogenesis begins before its implantation, unlike in human [20].

### 2.5. Menopause

Menopause is a human term, literally corresponding to the fact that menstruation stops when women get older. Therefore, this term is not literally applicable to other species, such as for horses. It corresponds, however, to ovarian dysregulation and final reduction of the oocyte pool, with a decrease starting during fetal period, and gradually after puberty. Such a biological process has also been described in horses [88,89]. Indeed, it has been estimated that mares reach ovarian senescence on average at 25 years old [20]. Moreover, around 17% of mares older than 20 do not ovulate any more [88,89]. In non-human mammalian species that have been studied, only mares seem to be affected by this process, partly because of the prolonged lifespan in this species.

## 3. Comparison of Assisted Reproduction Techniques in Horses and Humans 

### 3.1. Ovulation Stimulation

The stimulation of ovulation is routinely used in humans whereas it remains a poorly successful procedure in horses. One of the advantages of stimulation is that mature oocytes are obtained. The retrieval of immature oocytes from unstimulated follicles is widely used in equine and is also possible in humans, but live birth rates after in vitro maturation remain much lower in humans (see Section 3.3).

In humans, the first birth after IVF, Louise Brown, was reported in the UK in 1978 by R. Edwards and Pr. P. Steptoe [90]. In the early 1980s, births after IVF cycles, unstimulated or stimulated with clomiphene citrate, were reported in different countries. Given the low pregnancy rates, the use of urinary gonadotropins emerged in the USA [91] but results stayed not satisfactory. For this reason, the downregulation of endogenous gonadotropin synthesis resulting from the co-administration of gonadotropin-releasing hormone (GnRH) agonists was introduced in the late 1980s and rapidly became the standard of care. Timely induction of final oocyte maturation during the late follicular phase and prior to oocyte retrieval was induced by a single bolus dose of human chorionic gonadotropin (hCG). Commercial gonadotropins have diversified (urinary or recombinant) and stimulation protocols have evolved considerably with the use of GnRH agonists or antagonists and a trigger by antagonist or hCG. Subsequently, randomized controlled trials involving co-treatment with GnRH agonists and antagonists found similar IVF success rates, with a lower overall gonadotropin consumption and reduced rates of ovarian hyperstimulation (OHSS) [92,93]. In contrast, in horses, many treatments, including equine pituitary extracts (EPE), equine chorionic gonadotropin (eCG), gonadotropin releasing hormone (GnRH), as well as immunization against inhibin and partially purified equine FSH (eFSH) have been used with little success to try and superovulate mares [94]. More recently, recombinant equine FSH and LH have been demonstrated as able to increase ovulation rates and embryo recovery in mares, but defined, repeatable protocols still need to be developed [95]. Thus, in vitro oocyte maturation (IVM) becomes compulsory to increase in vitro embryo production from in vivo collected oocytes, as only one or two preovulatory follicles per cycle are available for puncture (see Section 3.2).

### 3.2. Oocyte Collection and Ovum Pick up (OPU)

To produce embryos in vitro, the first necessary step is oocyte collection. In women, collection from live donors is the only ethical way to access oocytes, whether from the infertile patient or an oocyte donor. This procedure is performed by transvaginal follicular puncture under ultrasonographic control in both species. In horses, oocytes can be collected from live or dead animals (i.e., most commonly, collection of ovaries at the slaughterhouse). Oocyte collection from a known donor is essential to control the full genetics of the in vitro produced embryos in animals. Like in women, OPU is performed for live animals. Moreover, in horses, many laboratories offer the service of oocyte collection and ICSI after the shipment of the ovaries of valuable mares that have died unexpectedly.

Transvaginal ultrasound-guided follicular aspiration was first described in humans in 1983 [96,97], shortly before the first transvaginal OPU was successfully achieved in horses [98]. This procedure is carried out routinely in human IVF [96,97] and veterinary laboratories for its simplicity and effectiveness. In humans, OPU follows ovarian stimulation and is scheduled between 34 and 37 h after the administration of hCG. It is performed as an outpatient procedure under conscious sedation with local epidural, spinal, or general anesthesia in women. In horses, follicular growth is monitored by ultrasound during the cycle, taking advantage of the follicular waves described above, to reach the optimal number of follicles >1 cm diameter to be punctured [99,100]. This allows for the recovery of 5–12 immature oocytes per OPU cases, whereas <1 is obtained when targeting only preovulatory follicles [100,101]. An important detail is that the oocyte in equine is deeply embedded in the follicular wall [102], making it necessary to repeatedly scrape off the follicular wall in order to retrieve the oocyte and lengthening the collection process [103].

The OPU procedure is generally very well tolerated in both women and mares [97,99,104,105,106]. In horses, it can be repeated every two weeks in routine management [106].

### 3.3. Oocyte In Vitro Maturation (IVM)

Mature, oocytes (metaphase II) together with matured cytoplasm are needed to achieve successful fertilization. In many mammalian species, including horse and human, oocytes collected from immature follicles cultured in vitro can progress to the metaphase II stage, using a procedure known as in vitro maturation (IVM), that takes from 24 to 38 h, depending on species.

Ironically, although the first human embryo generated in vitro was obtained from immature oocytes matured in vitro [107], this technique is still considered as experimental and is not the currently standard ART procedure. The use of this approach in the early days of reproductive medicine (first human live birth after IVM [108]; first live birth from a woman after IVM with her own oocytes in 1994 [109]) was justified to circumvent non-controlled stimulation protocols, timing of ovulation, and the difficulty of harvesting mature oocytes from large pre-ovulatory follicles. With the development of controlled ovarian stimulation (COS) protocols, the use of in vivo matured oocytes for fertilization became the gold standard. Because ovarian stimulation is not efficient in the horse, IVM is used routinely. Factors affecting oocyte maturation in vitro and in vivo in horses have been reviewed extensively [14,110]. In the horse, maturation media differ between laboratories [99,111]. It is important to note that, in horses, oocytes that have just started cumulus expansion are the most able to mature in vitro to metaphase II compared to oocytes with compact cumuli [103].

In humans, IVM indications have now been extended to patients with contraindication to ovarian stimulation, such as severe overstimulation syndrome in case of polycystic ovary syndrome (PCOS) [112,113] or in the case of hormone-dependent cancers before preserving fertility [114]. This alternative is also offered to some patients with poor ovarian reserve or in whom puncture performance after conventional IVF was low, even if the benefit of this strategy remains controversial [115,116,117]. More recently, IVM has been reported as the only therapeutic alternative for patients with FSH resistance syndrome for whom conventional IVF is totally ineffective [118]. It would seem that, depending on the genotype and/or phenotype of these patients, IVM could have a real place in the management of their infertility [119]. Nevertheless, even though IVM techniques have been improved, the lack of data comparing live births [120] or miscarriage rate [121] after IVM or standard IVF explain why the use of this technique remains relatively anecdotal (0.0003% in European ART centers) [122]. In horses, PCOS has not been reported and the horse could thus not be a spontaneous model for this disease.

### 3.4. Oocyte Manipulation

As explained above, mature oocytes are most frequently used in humans whereas immature oocytes are collected in horses to subsequently undergo IVM before fertilization.

In horses, transport of immature oocytes is performed preferably at 15–18 °C in buffered holding media using special packaging [99,123,124].

Both human and equine oocytes collected from dominant preovulatory follicles need to be kept at 37 °C to avoid damage [125]. It has been suggested that human microtubule spindles are thermosensitive [126] and that changes in temperature can irreversibly affect spindle integrity [127]. The meiotic spindle plays a major role in the successful completion of meiosis by controlling chromosomal movements throughout the stages of meiosis. Disturbances of meiotic spindles have been suggested as influencing chromosomal segregation and subsequently oocyte aneuploidy. Previous studies have shown in humans that the absence of the meiotic spindle is associated with poor fertilization rates and low embryo development [128]. Temperature fluctuations, which can easily occur in routine ART as well as in veterinary practice, may result in major abnormalities of chromosomal distribution.

Furthermore, human oocytes appear to have very limited ability to combat alkalosis. Therefore, pH (pHi) regulation or regulatory capacity may be impacted by in vitro culture conditions although there may be slight species variations in oocyte intracellular pH (reviewed by [129]). pH is known to affect elements of the actin cytoskeleton of the embryo and the oocyte cytoskeleton is responsible for meiotic spindle positioning. Interestingly, the surrounding cumulus cells transmit the ability to regulate pHi to the enclosed oocyte via gap junctions. As a result, denuded mature metaphase II oocytes are incapable of actively regulating pHi [129]. Therefore, in some clinical IVF procedures, such as intracytoplasmic sperm injection (ICSI) or pre-freezing oocyte denudation, in which cumulus cells are removed, the oocytes are extremely sensitive to pH disturbance up to several hours after fertilization.

The gamete intrafallopian transfer (GIFT) technique (transfer of both oocyte and sperm in the oviduct) was used for a long time to treat idiopathic infertility in women. The improvement of in vitro fertilization techniques with embryo transfer and intrauterine insemination, both with increased pregnancy rates and reduced risk of twin pregnancies, however, has made this technique obsolete and almost non-existent nowadays [130]. In horses, oocyte transfers or GIFT in oviducts of synchronized recipients were used as an alternative to embryo transfer for mares (and stallion) that had history of reproductive failures, i.e., failed to produce an embryo or pregnancy, with ovulatory problems, persistent uterine infection, or pyometra (reviewed in [121]). As in humans, however, with the progress made in ICSI, oocyte transfer or GIFT are no longer used [131,132,133], although this technique could still be of interest for research.

### 3.5. In Vitro Fertilization (IVF) and Intracytoplasmic Sperm Injection (ICSI)

In most species, including humans, in vitro fertilization (IVF) is the most standard procedure to produce in vitro embryos.

Since its introduction in ART in the 1990s [134], ICSI has been rapidly incorporated into routine human IVF laboratory practices. According to the European registry published in 2016 by ESHRE, the share of ICSI in the 2015 activity in the different European countries was 76.7%. Some countries, such as the Czech Republic and Moldova, now only perform this technique [122]. Some intractable indications for ICSI were clearly established from the beginning as the technique itself was developed to overcome the inefficiency of conventional IVF for severe male infertilities, such as severely impaired sperm characteristics, including severe oligospermia, necro or cryptozoospermia, severe asthenospermia or akinetospermia, or some severe teratozoospermia like globozoospermia. In some cases, it is used as a second resort, when there has been a history of fertilization failure in conventional IVF with normal sperm parameters. In addition, this method of fertilization has made possible the use of surgical sperm, particularly testicular spermatozoa, in cases of azoospermia, which affects 1% of men and 10–15% of infertile men [135]. Subsequently, the indications for ICSI in men have been extended to other couples not concerned with male infertility, but for whom ICSI fertilization has become compulsory because it is the only method compatible with the practices or techniques otherwise used in the management of couples, such as: (i) IVM and IVF after warming frozen oocytes (as in the case of oocyte donation) where the resulting denudation of the oocytes makes conventional IVF unfeasible; (ii) in case of preimplantation genetic testing (PGT) which could be performed in association with ART to analyze the DNA from embryos (cleavage stage or blastocyst) to determine genetic abnormalities [136], where ICSI is preferred to conventional IVF as it avoids sample contamination from cumulus cells, extraneous sperm attached to the zona pellucida, and non-decondensed sperm within blastomeres that can affect the accuracy of genetic analysis [137]; (iii) PGT performed after biopsy and embryonic cell sampling and for which the presence of spermatozoa attached to the oocyte results in contamination of the sample with paternal DNA. Finally, the ICSI technique may sometimes be the only one used in certain ART centers or certain countries for purely economic reasons, where the treatment of couples is expensive and where it is considered, rightly or wrongly, that it is not reasonable to take the risk of fertilization failure in IVF such as Middle Eastern countries [138]. However, some studies show that there is no benefit in performing ICSI compared to IVF in the absence of male factors [139] and even live birth rates are lower with ICSI than with IVF by 10% [140]. Consequently, some learned societies have issued recommendations to avoid excessive recourse to ICSI in the absence of male infertility factors [141].

In horses, the first and only two foals born after IVF were produced using a mature oocyte collected from a preovulatory follicle [98] followed by surgical transfer of the embryo in the oviduct, 24–60 h after fertilization [142]. Because equine sperm does not easily penetrate the zona pellucida of in vitro matured oocytes due to zona pellucida hardening and because the partial or total withdrawal of the zona pellucida results in polyspermy [143], ICSI is the preferred ART procedure in equine, when in vitro production is needed due to male or female infertility, or in the case of extremely limited sperm numbers. This procedure is performed by a rising but still limited number of laboratories throughout the world, thus requiring oocytes and frozen semen to be shipped to these structures. Detailed procedures have been described, with better outcomes being obtained using PiezoDrill [99,111,144]. 

### 3.6. Embryo Development and Transfer

In humans, embryo transfer is a response to a parental project and the embryo can be transferred in the days following the OPU (after OS or IVM) or after cryopreservation. Depending on the country, the embryo can be solely transferred in its genetic mother or, in some cases, in another woman, whether through embryo donation or as a surrogate mother. In contrast, embryo transfer in domestic mammals is a way to exchange the genetics of animals without moving live animals. It reduces costs and enables sanitary control. In the equine industry, the equine embryo business concerns both OPU-ICSI produced or in vivo recovered embryos throughout the world.

In humans, since the early days of in vitro embryo culture, many modifications have been made to culture systems to optimize the culture environment and increase the yield and number of embryos of good potential available for embryo transfer (ET). This has resulted in most laboratories now culturing embryos under conditions that allow a culture until the blastocyst stage and to obtain high potential embryos with improved overall pregnancy rates. At the same time, the methods for selecting embryos eligible for in utero transfer have become more efficient, which has made it possible to promote single embryo transfers and reduce the time to pregnancy and live birth.

Unlike humans, the equine embryo is often transferred in another mare than the genetic mother. In vivo embryo collections are thus commonly performed in equine. In the 1970s, in equine, firstly surgical and then nonsurgical embryo recovery procedures were developed [145,146]. Initially, embryo transfers were reserved for old infertile mares, but the technology was rapidly extended to mares in competition, mares who foaled late (so as to produce the next foals earlier in the following year) and pubertal two-year-old mares. In vivo, the horse embryo is collected in the uterus at the blastocyst stage (day 6–8 post ovulation). Both in vivo produced and in vitro produced equine embryos are transferred at the blastocyst stage.

Culture conditions are a major concern for embryologists. Indeed, even if the gametic potential has a significant impact on embryonic development, it is accepted that suboptimal culture conditions can be deleterious and decrease the chances of pregnancy by impacting the number of available embryos and the intrinsic developmental potential of the embryos up to the adult age [147]. In humans, an international consensus has recently begun to emerge [148,149]. Air quality in manipulating rooms is a major concern. The laboratory buildings must be designed to ensure an effective overpressure and air renewal while limiting polluting agents such as microorganisms but also volatile organic compounds [148]. Culture conditions (pH, CO_2_ and O_2_ pressure, temperature, culture media, hygrometry, and asepsia) are critical and can impact embryonic potential and modify epigenetic marks in the preimplantation embryo [150,151]. In horses, so far, there is no international consensus on embryo culture conditions and several methods have been used successfully since the 2000s (reviewed in [152]). In any case, one way to control the culture environment in both human and equine is to monitor key performance indicators, such as fertilization rate, cleavage rate, blastulation rate, and good potential embryos rate, for which thresholds and benchmark values have been described [153].

It is relatively easy to obtain a good development of embryos until the blastocyst stage in human, whereas stage 8–16 cells can be reached in the horse with a variety of in vitro conditions, but blastocyst formation is more difficult to obtain. Equine in vitro development is delayed compared to in vivo produced embryos: the 8–16 cells stage is only reached at four days post fertilization and the blastocyst stage between seven and 10 days post fertilization (see [154] for review). In addition, expanded blastocysts with a thin trophoblast layer and a distinct inner cell mass, such as in vivo produced blastocysts, cannot be obtained [154]. The zona pellucida does not get thinner and a full capsule surrounding the embryo is not produced in vitro [152,155]. After the transfer of in vitro produced equine embryos into recipient mares, however, the inner cell mass becomes apparent and a complete capsule is formed [156], demonstrating that embryo culture conditions are still suboptimal for equine development and more studies are required.

Another challenge both in human and horses is to select the embryo to be transferred fresh or to be frozen to reduce the time to achieve pregnancy while limiting the risk of discarding viable embryos from the attempt. In humans, in recent years, many tools have been developed to assist medical teams in this selection, while in horses, the quality assessment of cultured embryos is at its beginning. Methods can be invasive, such as embryo biopsy for pre-implantation genetic testing [124,157,158], or non-invasive, such as embryo monitoring by time-lapse imaging [159,160,161,162,163], metabolomics, or aneuploidy diagnosis on spent culture media (niPGT–A) [164]. These different techniques present different sensitivities, specificities, and acceptability, but have allowed to better control early embryonic development, to refine the criteria for the choice of the embryo to be transferred, and to promote single embryo transfer and thus considerably reduce the risks of multiple pregnancies (see below).

### 3.7. Embryo Biopsy

Genetic diagnosis of human embryos was initially developed and designed to avoid the transmission of serious X-linked diseases (as adrenoleukodystrophy, X-linked mental retardation and Duchenne muscular dystrophy). Embryo biopsies at the early cleavage stage were first performed from 1989, taking one or even blastomeres for preimplantation genetic testing (PGT) of all embryos on day 3. The first live birth following this manipulation was obtained in 1990 [165]. Nowadays, PGT might be reliably adopted to test human embryos from IVF cycles for monogenic diseases (PGT–M), chromosomal structural rearrangements (PGT–SR) and aneuploidies (PGT–A; previously also known as preimplantation genetic screening, PGS). The development of extended embryo culture until blastocyst stage has enabled to perform biopsies on day 5 or 6. Several trophectoderm cells are biopsied, thus leaving the ICM intact and significantly increasing the number of cells and consequently DNA quantity obtained for genetic analysis. The biopsy of the first polar body (PB) was also proposed to diagnose maternally transmitted monogenic diseases, later complemented by analysis of the 2nd PB obtained after fertilization [166]. This technique is less invasive and allows an additional two days for genetic analysis before reaching the day 3 stage and possibly performing a fresh embryo transfer. In the event of an inconclusive result, it is still possible to perform an embryo biopsy at day 3 or at the blastocyst stage.

In horses, embryo biopsy is performed in morulas and blastocysts, passing through the zone pellucida and collecting cells in the periphery of the embryo to avoid the inner cell mass that is not visible in morulas. For older embryos, a PiezoDrill is used to breach through the capsule and trophoblast cells are collected, far away from the inner cell mass [167]. This technique is widely used commercially for embryo sexing [124,168], as well as in genetic evaluation for embryo selection [111,169,170].

Nowadays, the improvement in the efficiency of high-throughput sequencing techniques with the next generation sequencing (NGS) in both species and, in the human, the improvement in blastocyst freezing/warming methods with the advent of vitrification has clearly contributed to the generalization of embryo biopsy both in humans and horses and the extension of their indications: improved pregnancy rates, reduction of the transmission of serious diseases in humans [171], but also sex determination and genetic evaluation in horses. The next challenges rely on the development and use of less technically challenging and/or non-invasive techniques to perform genetic testing both in humans and horses [111,172,173].

### 3.8. Oocyte and Embryo Cryopreservation

Cryopreservation programs are crucial to optimize ART protocols, safety, and efficiency. The greatest challenge during cryopreservation of embryos and oocytes is to prevent ice crystal formation and avoid cryoprotectant toxicity, main causes of cell death associated with cryobiology, while maintaining the cellular functions and viability of the embryo or oocyte [174]. In humans, embryo cryopreservation has decreased the number of fresh embryos transferred, and thus the occurrence of multiple pregnancy. Oocyte freezing has revolutionized fertility preservation, whereas in horses, embryo cryopreservation is commonly used for embryos produced in vitro but is still not efficient for in vivo produced embryos.

The first pregnancy and birth after transfer of frozen-thawed human embryos were reported in Australia in 1983 and 1984 [175,176], at the same time as the first successful cryopreservations resulting in live foals [177]. In both species, various protocols have been proposed using different types and concentrations of cryoprotectants, equilibration timing, cooling rates, and cryopreservation devices (reviewed in [123,178,179]). For almost 20 years, slow freezing was the only technique used in humans. In contrast, in horses, slow cooling was not widespread due to the poor success and the financial investment needed. Major factors affecting the success of cryopreservation of equine embryos are indeed their size, their large blastocoel and the glycoprotein capsule that reduces the penetration of cryoprotectants. Embryos smaller than 300 µm freeze well (pregnancy rates around 50–60%), while larger embryos result in poor pregnancy rates (around 20–30%), whether with slow cooling or vitrification (for review [179]). Thus, so far, most embryos are transferred fresh. Nevertheless, the recipient mare may be far away from the laboratory, so embryos are shipped all over the world in specially designed cooling containers. Successful procedures for equine embryo cooling were developed in 1987 [180] and are still effective, enabling successful transfer within 24 h of collection.

Vitrification, that allows the solidification of cells into a glass-like state without the formation of ice, has supplanted slow freezing in humans over the last ten years and is increasingly used in horses. In humans, available data suggest that vitrification/warming is superior to slow freezing/thawing with respect to clinical outcomes and cryosurvival rates either for oocytes, cleavage-stage embryos and blastocysts (reviewed in [181]). The high results of vitrification, particularly at blastocyst stage, have made it possible to change clinical practices with differed embryo transfer (freeze-all strategy) to avoid ovarian hyperstimulation syndrome with identical to superior results [182]. In equine, vitrification has proven very successful for in vitro produced embryos that are smaller than in vivo embryos and is now widely used, even though these embryos can also be cryopreserved with the slow cooling method [183,184]. 

Procedures aiming at collapsing the blastocyst before vitrification through blastocoel micro-aspiration appear to improve post-thawing pregnancy rates in humans [185] as well as in horses where post-thawing pregnancy rates of embryos larger than 300µm were similar to that for embryos smaller than 300 µm [183,186]. In humans, blastocyst collapse could be routinely performed in IVF laboratories using a laser or blastocoel micro-aspiration but as with the horse, the results are controversial, and this technique involves additional handling. However, this method requires a micromanipulator, which is too expensive and too complicated to become widespread in the equine embryo transfer industry. Very recently good pregnancy rates were obtained with >300 µm embryos that were vitrified after manual blastocoel puncture [187]. If this is confirmed, this technique can be used on a larger scale and, unlike humans and other ruminants, the freezing of equine embryos may develop rapidly.

In humans, the cryopreservation of embryos has raised ethical, moral, and legal questions. In addition, the embryos belong to the couple, which can be problematic in case of separation. Therefore, some countries such as Italy have widely developed oocyte cryopreservation as an alternative to previous ban on freezing embryos.

Oocytes are more sensitive to freezing and thawing procedures and the use of vitrification has considerably improved the results (reviewed in [181]), allowing ART center to largely proposed oocyte cryopreservation in fertility preservation programs. Despite enormous progress made in vitrification techniques, oocytes remain the cells mostly exposed to damage in the freezing/thawing process when compared to embryos and spermatozoa [188,189]. For example, the volume to surface ratio of the oocyte is greater than other cells, thus complicating the dehydration process. Moreover, membrane characteristics and permeability differences appear to make the oocyte more sensitive to chilling injury. It is well known that oocyte cryopreservation induces premature cortical granule release, causing zona hardening that interferes with fertilization, requiring the use of ICSI. Additionally, due to the high rates of oocyte derived human aneuploidy, alterations to the oocyte meiotic spindle in response to low temperatures from cryopreservation are concerning with regard to potential impact on chromosome remodeling [190]. In horses, the first foals born after oocyte cryopreservation were reported in 2002 [191], but only 14% of pregnancy rates were obtained after oocyte transfer in the oviduct of recipient mares. More recently, after oocyte vitrification, only a few blastocysts were obtained and resulted in an even smaller number of foals [192,193]. It has been shown that, in equine, vitrification could lead to aberrant spindle configuration, leading to poor chromosome alignment [192,194]. As OPU is well established in horses and oocyte shipment is in general necessary, the cryopreservation of equine oocytes before ICSI is a promising technique which requires more studies. Indeed, the first foal born after oocyte vitrification and ICSI has been reported recently [195].

## 4. Effects of Maternal Environment in Equine and Human

To date, in humans, it has been clearly admitted that advanced maternal age, obesity, excessive sport practice, and in contrast, a sedentary lifestyle, alcohol, and tobacco, have a clearly negative effect on fertility with an extended time to pregnancy. Some maternal environments also are problematic in horses, such as maternal aging, obesity, or excessive sport.

### 4.1. Effects of Maternal Age

Age is a major problem in humans, as the delay for conception gradually increases with time for women, for whom, aging is a challenge for conception. Horses are facing the same issues about maternal aging. Indeed, as women who have a professional career, mares have a sporting career before producing foals. As in humans, fertility decreases with age [196,197]. It was calculated that if a 10% reduction in fertility was used as a threshold for keeping a mare in breeding programs, mares older than nine years should not be conserved [197]. Thus, the choice of ART in both species is related to maternal aging. In the equine industry, OPU–ICSI–ET is essentially used in older mares, with embryo transfer in younger, healthy recipient mares so as to reduce age-related potential effects [198].

In horses, reproductive tract dysfunction [199], reduced ovarian reserve [89,200], and decreased oocyte quality [201,202,203,204] have been well identified as age-related factors affecting fertility. Reproductive tract degeneration occurs gradually as mares grow older with oviduct lumps [81] and uterine cysts being frequently observed in old mares. Both are thought to impair oocyte and embryo movement and therefore reduce fertility. In relation to mares’ parity, severe endometrial fibrosis is also observed [199,205,206], together with increased vessel elastosis [207,208] and several deleterious histological modifications [199,209].

In humans, 50% of fertilized embryos do not implant and most of the implantation failures are mainly due to aneuploidy and these abnormalities are predominantly oocyte-related, since between 20% and 30% of human oocytes have defects of this type [210] and maternal aging is the only etiologic factor unequivocally linked to embryo aneuploidy [211]. In contrast, in horses, in vivo fertilization rates are >90% regardless of maternal age but when in vivo embryos from old mares are transferred to young recipient mares, pregnancy rates are lower [67,212]. Oocyte quality is negatively associated with maternal age [213,214] and fewer oocytes reach the second metaphase during in vitro maturation when mares are old [215]. Nevertheless, similar mechanisms are altered by maternal aging: aneuploidy, with age-related chromosome misalignment on the metaphase II spindle [201], is the most visible biological process affected by the age-related oocyte quality drop in humans and in horses [216,217]. In both species, oocyte aneuploidy is mainly due to a premature separation of sister chromatids at the end of the prophase I [210,216,218]. Moreover, for both species and unlike rodents, the meiotic spindle assembles in a slow and unstable way and in equine, maternal age predisposes to more spindle instability, which can partly explain segregation errors [219].

Recently, an impact of age has been reported on single human oocyte transcriptome [220]. In particular, the expression of genes related to mitochondrial activity was reduced [221]. This provides arguments to use mitochondrial transfer to improve IVF pregnancy rates in elderly women [222], even if more data are necessary before an eventual implementation. Globally, age could impact all proteins [223,224] and aneuploidy is only the most accessible defect detected using conventional technologies. In horses, to the author’s knowledge, high throughput analysis of the oocyte is not yet available. The expression of 48 genes was analyzed in cumulus–oocyte complexes according to maternal age and showed an age-related differential expression associated with lower quality and reduced developmental competency in oocytes from old mares [204]. In vivo, the expression of a limited number of genes required for follicular and oocyte maturation was studied in the cumulus cells and the oocyte, showing asynchronous expression in old compared to young mares [202]. The addition of follicular fluid from young or old mares to in vitro oocyte maturation medium, however, did not influence oocytes maturation nor developmental competence [225]. Like humans, mitochondrial function is also perturbed by mares’ age. Indeed, a temporal reduction of mtDNA copy numbers was described in oocytes from old mares during in vivo [202] and in vitro [203] maturation. As compared to younger controls, the oocyte mitochondria of old mares (>12 y.o.) were more swollen and had lost transverse cristae after in vitro maturation, suggesting mitochondrial degeneration during the IVM process [203]. The age-related negative effects observed in oocytes are also found in embryos. Mitochondrial DNA is less abundant in in vitro produced blastocysts when the oocyte has been collected in an old mare [226]. In addition, one study, using RNA sequencing of equine day 8 blastocysts, showed that maternal age disturbs mitosis, translation and transcription, cell signaling and adhesion, mitochondrial function, and cell fate commitment [227].

Finally, age has been associated with de novo mutations. First described in men, for whom an increased incidence of achondroplasia has been observed according to age [228]), there is growing evidence that it may concern both sexes [229]. With age, an accumulation of de novo mutations occurred at spermatogonial steps due to the continual renewal of the stem cells pool by mitosis [230]. Such mechanisms also explain the increased incidence of cancer according to age. In women, increased incidence of autism and schizophrenia has been observed in offspring born to elderly parents, pathologies which are known to be associated with de novo mutation [229]. So far, there is no such study in horses.

### 4.2. Effects of Maternal Obesity

The prevalence of obesity is currently close to 15% of the general population in humans [231], with more than half of women of reproductive age being overweight (BMI ≥ 25 kg/m^2^) [232]. Obesity is also a growing concern in horses, probably in relation to the fact that horses are increasingly being regarded as pets. Depending on the location and use of the horses, 2–72% are considered as overweight and 1–19% as obese [13,14,233,234,235,236], with the lowest incidence observed in athletic horses involved in competition. Associated with obesity, the equine metabolic syndrome (EMS) [237] is comparable to the human metabolic syndrome [238].

In humans, female obesity has been clearly associated with several adverse pregnancy outcomes [239], including risk factors for infertility whatever the menstrual cycles or smoking habits, with a higher risk in primiparous women [240]. This phenomenon seems to be mainly due to anovulation [241]. In contrast, in mares, obesity and insulin resistance have been associated with altered estrous cycles [15,242], reduction or loss of anestrus in winter [243,244], and increased incidence of LUF [15,242], but others did not report any difference on ovarian activity nor estrous cycle according to maternal BCS [245].

In women, decreased oocyte retrieval, reduced oocyte quality, and reduced rates of pre-implantation embryo development are generally observed [246] as well as poorer IVF outcomes and a decreased probability of live birth following IVF, worsened when associated with polycystic ovary syndrome [247]. In addition, when higher doses of gonadotropins are used, there is an increased risk of cycle cancellation or miscarriage [246]. Recently, using proteomic analysis, it was shown that granulosa cells mitochondria were damaged and that the endoplasmic reticulum stress response was accompanied by dysregulated hormonal synthesis [248]. These alterations may be related to the high free fatty acid and triglyceride levels detected in human follicular fluid [248] and altogether indicate a poor oocyte quality.

Even though fertility does not seem to be very affected, mainly due to the relative lack of extreme obesity in horses, the obese mare shares a lot of similarities with the obese woman. Insulin resistance, hyperinsulinemia [15,242,249] and several hormonal changes, such as increased plasma leptin, insulin-like growth factor 1, prolactin, and decreased plasmatic thyroxine, are related to obesity in mares [15,243,249]. Leptin has been suggested to support luteal establishment in a dose-dependent way in equine [116]. Moreover, as in humans, equine obesity is associated with increased inflammatory factors [250]. The uterus appears to secrete more inflammatory molecules before implantation when mares are insulin resistant compared to control mares [251]. In addition, the composition of follicular fluid in metabolic hormones and cytokines of follicles from mares with EMS or only obese are altered [252,253]. EMS and obesity also only alter expression of genes related to lipid homeostasis, endoplasmic reticulum stress, and mitochondrial function in granulosa and cumulus cells [252,253]. Moreover, the lipid composition of oocytes and embryos is perturbed by maternal obesity [249,253], suggesting reduced oocyte quality. In embryos, the expression of genes related to inflammation, lipid homeostasis, endoplasmic reticulum, as well as oxidative and mitochondrial stress is also altered by maternal obesity [249].

### 4.3. Effects of Maternal Excess Sport

Underweight (body mass index (BMI) below 18.5 kg/m^2^) is associated with an increased risk of anovulatory infertility.

In humans, the main aetiologies are (1) eating disorders that impair female fecundity due to the ovulatory dysfunction and a reduction in sexual activity, (2) energy deficiency mainly due to excess sport practice and that mainly concerns female athletes, (3) lipodystrophies, a heterogeneous group of rare disorders, characterized by selective loss of adipose tissue in the absence of nutritional deprivation or catabolic state, (4) starvation and undernutrition due to the self-imposed dietary restriction in developed countries, or malnutrition in developing countries, and (5) a few chronic systemic diseases [254]. Regarding physically active women, a medical triad is observed associating low energy availability with or without disordered eating, menstrual dysfunction, and low bone density. Health is a prerequisite for optimal performance, and menstruation is often considered as a problem for most of them, with either a negative or neutral impact on performance [255]. Although a systematic review and meta-analysis indicated that exercise performance might be trivially reduced during the early follicular phase, they concluded that a personalized approach should be taken, based on everyone’s response to exercise performance across the menstrual cycle. So, even if there is no clear evidence for a relation between performance and menstrual cycle, hormonal medication for both contraception and to manipulate the menstrual cycle is very commonly used [256]. Based on a cohort of elite female athletes, oligomenorrhea/amenorrhea has been reported to be higher in athletes without hormonal contraception. Stress fractures and iron deficiency, common for these women, are associated with oligomenorrhoea/amenorrhea/menorrhagia and eating disorders. In addition, in this cohort, 15% of these women were engaged in disordered eating practices in order to conform to gender ideals [257]. Whatever the consequence of such practice, a recent study, comparing a few elite athletes to active controls, reported that regularly physically active (>150 min per week) elite athletes get pregnant easily and deliver healthy babies [258].

Apart from being underweight, associated with anovulation and metabolic disorders, it seems that elite female athletes are not particularly infertile. However, in general, it is necessary to stop training and competing for a long period to have a child, and many of them decide to delay pregnancy. Oocyte preservation could be an opportunity for these women.

In the equine industry, ART, especially embryo transfer, is very successful in competing horses because it enables breeders to produce foals when mares are still performing. Effects of sport on reproductive outcomes in horses have been related to a higher internal temperature in exercising mares which can be deleterious for reproduction [259]. Indeed, altered ovarian follicle as well as endocrine (cortisol and LH) dynamics are observed in exercising mares [259,260]. Effects of sports activity on embryo quality and recovery rates are controversial as reduced embryo recovery rates [259,261], with poor embryo quality [259] have been reported by some in exercising mares while others did not observe any deleterious effect [262]. The latter observation could be due to better cycle monitoring that has been shown to improve embryo recovery efficiency in exercising mares [263]. Depending on breed, racing after insemination/mating has been shown to be deleterious for foal production [264], but moderate exercise did not influence pregnancy outcomes [265]. Furthermore, one study showed that retired performance mares have a much higher incidence of glandular differentiation disorders related to ovarian dysfunction [266] compared to non performing controls.

## 5. Where Is the Mare a Good Model for ART in Humans?

Overall comparisons between the physiological and ART specificities of woman and mare are shown in Table 1. Contrary to laboratory models, the horse is a long lived monocotous animal. Its high economical and sentimental value to humans justifies its use in reproduction until old age and the investment in state-of-the-art ART to overcome infertility and to increase foal production. As seen in Table 1, there are many similarities but also large differences in the physiology, ART and effects of environment between humans and horses. Yet, the horse may be an excellent model, offering possibilities not enabled by other models to study ART for humans. Its large size also enables repeated sampling that is not allowed by smaller models. Alternatively, the human may be also a very valuable source of inspiration for scientists and veterinarians working with horses. 

### 5.1. Developmental Origins of Health and Disease

In the past, ART has been associated in other species with adverse effects during pregnancy, e.g., with the onset of large offspring syndrome in cattle [267]. In addition, periconceptional environment and ART are now known to affect long term offspring phenotype through the establishment of epigenetic marks in the early stages of development [268]. Although valuable information on these effects is collected using rodent and other models [147], large monocotous animals with a long lifespan supported by a sports industry and for which ART is used with similar objectives as in humans can bring invaluable information on the long-term effects of ART. Using horses of known genetic background, health and performance comparisons between offspring produced by natural mating, artificial insemination or ART could be organized, whether prospectively or retrospectively through epidemiological studies, as performance and genealogy is recorded by studbooks of national organizations such as the French institute of horse and horse riding (IFCE) in France. The horse, with a completely sequenced genome (×6.8 in 2021) [269] is also a valuable model to look at differences in gene expression in embryos. Moreover, its size enables embryo bisection and subsequent differential analysis of gene expression in the inner cell mass and the trophoblast [227], which could bring important insight into mechanisms.

### 5.2. Follicular Cycle

With a very long follicular phase, during which the mare accepts breeding, the mare is unique among domestic animals. A long follicular phase is also present in women. Thus, the mare could be a very good model for studying the regulation of follicular growth. Because of the particular structure of the equine ovary and its ovulation fossa, and because of the large follicular size, experimental sampling of follicular fluids throughout the cycle can be performed to study follicular maturation, without rupturing the follicle [270]. Complete removal of follicular fluid from a preovulatory follicle was used to demonstrate that follicular fluid was not necessary for ovulation [271]. The same approach enables the injection into the follicle of experimental compounds, immature oocytes to perform an “ex vivo maturation” [272], or spermatozoa. In addition, the destruction of follicles through ultrasound-guided follicular puncture starts a new follicular wave and enables an exact timing of follicular growth. Finally, with the occurrence of LUF in both equine and human, the mare has thus been suggested as a potential model for investigating anovulatory infertility in women [273].

### 5.3. Oocyte Retention in the Oviduct

The mare is unique in that unfertilized oocytes remain in the oviduct. Given that most ectopic pregnancies in women occur in the oviduct [274], it might be interesting to use the mare model to study factor involved leading to the embryo-driven movement in the oviduct. For example, topical PGE2 application to the oviduct has been shown to induce oviductal patency [275].

### 5.4. Oocyte In Vitro Maturation (IVM)

As mentioned above, IVM is only used in a few clinical situations in humans and thus, IVM conditions have not been improved for many years. Since IVM is almost compulsory in the equine species, the horse could be a good model for humans to work on IVM conditions.

### 5.5. Effects of Environment: Aging, Obesity and Excess Sport

The horse is a species where animals are bred at an old age, with breeders that continue breeding as long as they can produce a foal from their favorite mare(s). It is thus common to see nulliparous mares (mares that did not previously foal, also known as maiden mares) bred at old age. Indeed, nulliparous mares older than 10 years of age have been shown to represent 20.5% of Finn horse and 15.5% of Standardbred bred mares in Finland [276]. Conversely, many old mares had had a large number of foals. Thus, the mare has been proposed as a model for oocyte aging, especially since similar mechanisms are involved in both species, including a high incidence of aneuploidy and mitochondrial defects [10,213].

As mentioned in Section 4.2, excess BMI (overweight and obesity) is frequent in horses, although morbid obesity is extremely rare. The incidence of the equine metabolic syndrome also increases in the equine population, as well as in the human population. Deleterious effects of excess body weight on oocyte quality, follicular cycles, and reproductive tract inflammation are shared between the two species. In addition, obesity and the metabolic syndrome are often associated with aging in both species.

Since progress in understanding and preventing the effects of aging and obesity on oocyte would benefit both species, research in this area could be co-funded by medical and veterinary research.

In terms of excess sport, the intense training of thoroughbred mares in their early age and their very thin body condition at this stage could also be used to model effects of excess sport in young women. In particular, long-term consequences on reproductive outcomes and epigenetic effects could be followed up in horses.

### 5.6. Where Is the Mare Definitely Not a Good Model for ART in Humans?

In terms of physiology, the first birth of IVF foals [142] was never reproduced in horses and only ICSI is currently used for fertilization of equine oocytes [111]. Thus, the horse cannot be used as a model for human IVF. In addition, the horse uterus is not able to sustain twin pregnancies, in contrast to humans.

As mentioned before, PCOS is not a spontaneous pathology known in horses, and thus horses cannot be used as models for this human disease, unless it could be induced by exogenous treatment or genetic selection as already done in other species [277,278].

### 5.7. Where Can the Woman Help the Mare?

Many ARTs are used in women that are not mastered in horses. In particular, embryo and oocyte cryopreservation need to be improved in mares and experience from human biologists is certainly of great help.

## 6. Conclusions

In conclusion, although there are large differences between horses and humans in terms of reproductive anatomy, physiology, and ART, both species share infertility concerns due to ageing and obesity. Some techniques have been mastered in humans but not in horses, such as superovulation, as well as embryo and oocyte cryopreservation, whereas others are more advanced in horses, such as IVM. Thus, reciprocal knowledge could be gained through the comparative study of ART and infertility treatments both in women and mares, even though the horse could not be used as a single model for human ART.

Women and mares both ovulate a metaphase II oocyte (1). In both species, once fertilization is achieved, the oocyte resumes its meiosis (2). Extrusion of the second polar body happens quickly after fertilization and is followed by the formation of the female and male pronuclei (PN). In women, PN fading is observable 23 ± 1 h post insemination (HPI). Then, both pronuclei merge to form the nucleus. By 27 ± 1 hpi in women and 24 h in mares, the first cleavage occurs (3). The second division takes place over the next 24 h in both species (4) and the 8-cell stage is reached within 3 days post fertilization (5). In women, this is the last developmental stage in the fallopian tube. During the next day, a morula with at least 16 cells is formed (6). In horses, this is the last stage of development in the oviduct. The morula produces prostaglandin E2, that relaxes the muscles of the isthmus of the oviduct (utero–tubal junction) and enable the passage of the embryo into the uterus. Development to the blastocyst stage (6) is a little longer in horses than in humans (reached at 116 ± 2 HPI in humans and 7 days in horses). This is the last common developmental stage between the two species.

## Figures and Tables

**Table 1:** Summary of similarities and differences in periconceptional physiology and ART procedures between the woman and the mare. LUF: Luteinized Unovulatory Follicle; OPU: Ovum Pick-up; PCOS: Polycystic ovary syndrome.

		Woman	Mare
Similarities	Physiology	Formation of primordial follicles during fetal life	
		Two or three follicular waves per ovarian cycle	
		Mono-ovulatory cycles, LUF syndrome	
		Timing of early developmental stages until the blastocyst stage	
		Easy and efficient in vitro embryo culture up to the blastocyst stage in humans, a bit less efficient in horses	
	ART procedure	ICSI is widely used in both species	
		Blastocoel collapsing is used for freezing embryos	
		Development of several tools for embryo selection in ART procedures, including embryo biopsy and time-lapse	
		Shared interest in non-invasive preimplantation genetic testing	
	Impact of environment	Reduction of fertility with age	
		Frequency of embryonic aneuploidies	
		Menopause for woman; ovarian senescence for mares	
		Potential impact of excess sport on spontaneous reproduction	
		Effects of obesity on cycles, inflammation, follicular fatty acids and triglycerides, lower oocyte and embryo quality	
Differences	Physiology	Preovulatory follicles: 2 cm diameter	Large follicles (×2.1 human)
		ZP surrounding the embryo	Presence of a capsule in addition to the ZP
		Uterine prostaglandin secretion not needed for luteolysis	Uterine prostaglandin secretion needed for luteolysis
		Human blastocyst only reaches a maximum size of 200 μm and a few hundred cells before implantation	Horse blastocyst is larger on Day 7 (200–800 μm)
		Hatching and implantation on Day 7–10	No hatching, implantation on Days 35–40
		PCOS	No PCOS
	ART procedure	Oocyte collection limited to OPU OPU mostly performed after ovarian stimulation	Oocytes can be collected from live (by OPU) or dead mares OPU only performed in non-stimulated mares Oocyte embedded in the follicular wall making it necessary to scrape-off the follicular wall
		Possibility of ovarian stimulation, IVM, IVF, ICSI	Possibility of only IVM and ICSI; IVF not efficient
		Effective embryo freezing at all stages of development	Effective embryo freezing on embryos < 300 µm in size and in vitro produced embryos; methods need to be improved
		Oocyte vitrification is mastered	The technique of oocyte freezing needs to be improved and is not used commercially

## Data Availability

Data sharing not applicable.
